# Functional Analysis of Bna-miR399c-*PHO2* Regulatory Module Involved in Phosphorus Stress in *Brassica napus*

**DOI:** 10.3390/life13020310

**Published:** 2023-01-22

**Authors:** Kun Du, Yang Yang, Jinping Li, Ming Wang, Jinjin Jiang, Jian Wu, Yujie Fang, Yang Xiang, Youping Wang

**Affiliations:** 1 Jiangsu Provincial Key Laboratory of Crop Genetics and Physiology, Yangzhou University, Yangzhou 225009, China; 2 Guizhou Rapeseed Institute, Guizhou Academy of Agricultural Sciences, Guiyang 550008, China

**Keywords:** *Brassica napus*, miR399, PHO2, low phosphorus stress

## Abstract

Phosphorus stress is one of the important factors restricting plant growth and development, and the microRNA (miRNA) family is involved in the regulation of the response to plant nutrient stress by repressing the expression of target genes at the post-transcriptional or translational level. miR399 is involved in the transportation of phosphate in multiple plants by improving tolerance to low Pi conditions. However, the effect of miR399 on the response of low Pi stress in rapeseed (*Brassica napus* L.) is unclear. The present study showed a significant increase in taproot length and lateral root number of plants overexpressing Bna-miR399c, while the biomass and Pi accumulation in shoots and roots increased, and the anthocyanin content decreased and chlorophyll content improved under low Pi stress. The results illustrate that Bna-miR399c could enhance the uptake and transportation of Pi in soil, thus making B. napus more tolerant to low Pi stress. Furthermore, we confirmed that *BnPHO2* is one of the targets of Bna-miR399c, and the rejection of Pi in rapeseed seedlings increased due to the overexpression of *BnPHO2*. Hence, we suggest that miR399c-*PHO2* module can effectively regulate the homeostasis of Pi in *B. napus*. Our study can also provide the theoretical basis for germplasm innovation and the design of intelligent crops with low nutrient input and high yield to achieve the dual objectives of income and yield increase and environmental protection in *B. napus*.

## 1. Introduction

Phosphorous (P) is one of the mineral nutrients essential for plant growth, development, and reproduction [1]. Inorganic phosphate (Pi) is the predominant form of P directly absorbed by plant roots and a major transported form of P within plants [2]. Due to its low availability and slow rate of diffusion in soil, Pi availability is one of the main environmental constraints to crop productivity worldwide [3,4].

The application of P fertilizers has been the main method to maintain or improve crop growth for decades. However, P fertilizer is a non-renewable resource, with rising production costs, and serious environmental problems such as soil pollution and water eutrophication are caused by its large-scale application [5,6,7]. Therefore, in order to reduce the use of P fertilizer and promote sustainable agricultural development, it is necessary to be aware of the regulatory strategies of Pi homeostasis in plants that could provide a scientific framework for breeding new crop varieties with high efficiency in P acquisition and utilization.

In P-deficient conditions, on the one hand, plants increase P acquisition by optimizing P utilization, including genetic, physiological, biochemical, and morphological changes [8,9]; on the other hand, plants are more adaptable to low P environments [10] since some relevant macromolecules are degraded, and in turn, metabolic pathways are changed. microRNAs (miRNAs) are a class of non-coding small molecule RNAs widely found in eukaryotes that influence the activity of target genes by forming the RNA-induced silencing complex (RISC) at the post-transcriptional or translational level [11]. miRNA-mediated gene silencing can be involved in regulating the processes by which plants response to biotic or abiotic stresses [12,13,14]. In *Arabidopsis*, miR399 was the first miRNA that was proved to be associated with Pi limitation in plants, and also one of the miRNAs highly upregulated in expression during Pi starvation. The *Arabidopsis* miR399 family contains six members (miR399a–f), which are synthesized by the induction of the plant roots with a swift transmission to shoots after Pi uptake [15]. Among the seven family members of rice miR399, Osa-miR399d, f, and j were inductively expressed while the other members were induced to accumulate in the shoots in Pi-deficient conditions, indicating that each miR399 member may possess a different biological function [16].

miR399 target gene *PHO2* (*PHOSPHATE2*) encodes an E2 ubiquitin-binding enzyme and directs the cleavage of *PHO2*, which in turn is involved in the ubiquitinated phosphate transporters PHT1 (PHOSPHATE TRANSPORTER 1) and PHF1 (PHOSPHATE TRANSPORTER TRAFFIC FACILITA TOR 1), and so on, to maintain Pi homeostasis in plants [17]. Overexpression of miR399 in *Arabidopsis* accumulates excessive Pi in leaves, and symptoms of Pi toxicity similar to those of the *Arabidopsis pho2* mutant are expressed [2,18]. Studies in rice, wheat, and soybean have shown that miR399 can be specifically induced by Pi deficiency and that it inhibits the degradation of the Pi transporter protein PHO1 as well as the PHT1 family by mediating the cleavage of PHO2, thus enhancing Pi acquisition, translocation, and xylem Pi loading during P deficiency, also helping plants to accumulate sufficient Pi [19,20,21].

Rapeseed (*Brassica napus* L.) is one of the most important oil crops worldwide. It was originally produced in the Mediterranean region of Europe, and is now cultivated in large volumes in China, Canada, the USA, India, European countries, and Russia. *B. napus* has become an important source of edible oil, protein feed, and important raw industrial materials. Previous studies found that there are three members (a–c) of the miR399 family in *B. napus*, among which the mature sequences of Bna-miR399a/b members are identical and Bna-miR399c differs from them by one base [22,23], and its precursor sequence is highly conserved with *Arabidopsis*, rice, soybean, maize, and sorghum species (http://weblogo.berkeley.edu/logo.cgi, accessed on 10 September 2022). Our previous study showed that only Bna-miR399c was strongly induced to be expressed under low Pi conditions, suggesting that it might be involved in the response of rapeseed to a low Pi environment. Accordingly, this study was conducted to investigate the biological functions of Bna-miR399c in response to low Pi, mainly at the physiological, biochemical, and molecular levels, to provide a basis for improving the ability to uptake and transport Pi in *B. napus* and breeding new varieties of low-Pi-tolerant rapeseed.

## 2. Materials and Methods

### 2.1. Plant Materials, Growth Conditions, and Stress Treatments

The *B. napus* line J9712 (J9) was used as the explant for the genetic transformation.

Assessment of low Pi tolerance in shoots of *B. napus* at seedling stage: The T_2_ generation of Bna-miR399c-overexpressing (OE-Bna-miR399c) transgenic seeds and J9 seeds were germinated on wet filter paper. The germinated seeds with similar growth potential were then transferred to vermiculite medium containing 1 μM Pi Hoagland nutrient solution in greenhouse (22 °C, 16 h light/20 °C 8 h dark cycles) for low Pi treatment, and leaves and roots were taken after 15 d to detect relevant physiological indicators and gene expression levels.

Assessment of low Pi tolerance in roots of *B. napus* at seedling stage: The T_2_ generation of OE-Bna-miR399c transgenic seeds and J9 seeds were sterilized and germinated on 1/2Murashige-Skoog (MS) medium for 3–5 days. Seedlings with similar growth potential were selected and transferred to 1 mM Pi (normal Pi) and 1 μM Pi (low Pi) solid medium for sterile culture separately, then cultured vertically under 22 °C, 16 h light/20 °C, 8 h dark cycles. Root length, fresh weight, dry weight, and number of lateral roots were measured after 7 days of treatment. Each experiment was performed with three biological replicates.

*Nicotiana benthamiana* were grown in a light incubator (22 °C, 16 h light/8 h dark), and the 4-week-old plants were used for transient expression and dual-luciferase assays [24].

### 2.2. Constructs and Generation of Transgenic Plants

The precursor sequence of Bna-miR399c and the full-length CDS sequence of target gene *BnPHO2* were amplified from the *Darmor-bzh* cDNA, then inserted into the pMDC83 vector. The recombinant plant expression vectors pMDC83-Bna-miR399c and pMDC83-*BnPHO2* were introduced into *Agrobacterium tumefaciens* strain GV3101 for genetic transformation in *B. napus* [25]. Afterwards, transgenic lines were selected with Hygromycin B and confirmed by PCR amplification.

### 2.3. Gene Expression Analysis

Total RNA isolation was performed with RNA isolator Total RNA Extraction Reagent (Vazyme, Nanjing, China). Reverse transcription was performed using miRcute Plus miRNA First-Strand full-length cDNA Kit (TIANGEN, Beijing, China), and qRT-PCR was carried out using miRcute Plus miRNA qPCR Kit (TIAN-GEN, Beijing, China) on the StepOnePlus™ Real-Time PCR System (Applied Biosystems) [26]. The forward primer was the mature sequence of Bna-miR399c, and the reverse primer was the universal Primer Reverse provided in the kit. 5.8S rRNA was used as an internal reference. For PCR, the amplification program consisted of an initial 95 °C for 15 min, 45 cycles, followed by 94 °C for 20 s, 60 °C for 34 s. To detect the expression of the *BnPHO2* gene and phosphorus transport-related genes, qRT-PCR was performed by 2× AceQ Universal SYBR qPCR Master Mix (Vazyme, Nanjing, China). *BnActin* was used as the internal control. The 2^−ΔΔCt^ method was used to calculate the relative expression level [27]. The primers used for qRT-PCR are listed in Table S1. Each experiment was technically repeated three times.

### 2.4. Target Prediction and Validation

The Bna-miR1399c target gene was predicted online by psRNATarget (http://plantgrn.noble.org/psRNATarget/, accessed on 10 September 2022) [28]. Dual-luciferase assays were carried out to validate the target gene of miR399c. The fragments of potential miR399c target sites predicted online were constructed onto the backbone vector PGrDL-spb (Addgene) [29], and the corresponding dual-luciferase vector plasmids were constructed and transformed into *Agrobacteria* GV3101 (psoup) with the reverse complementary sequence PM (perfect match) of the mature sequence of Bna-miR399c as a positive control and the mutated form of the target sequence as a negative control. *Agrobacteria* containing individual constructs were suspended using the infiltration medium to a final concentration of OD_600_ = 0.7. *Agrobacteria* suspensions were mixed with a 10:1 volume ratio. Strains were slowly infiltrated into the abaxial side of leaves of *N. benthamiana* plants using a 1 mL syringe to cover the whole leaf. The plants were then grown in the dark for 2 days until further analysis. Dual-luciferase reporter assays (firefly luciferase and renilla luciferase) were prepared using Duo-Lite™ Luciferase Assay System kit (Vazyme, Nanjing, China) according to the manufacturer’s instructions. The activity of luciferase was quantified using a TECAN automated analyzer (Spark, Switzerland). The experiment was performed using three independent biological replicates. Data analysis was based on the methods previously reported [30].

### 2.5. Characterization of BnPHO2 Protein

The full-length CDS of *BnPHO2* without the termination codon (TGA) was PCR-amplified and inserted into the pMDC83-GFP vector. The NLS fused with RFP was co-expressed as a nucleus marker [31]. *Agrobacteria* containing the GFP:BnPHO2 fusion, P19 construct, and NLS-RFP were mixed at an equal volume, and injected into the abaxial epidermis of *N. benthamiana* leaves in the dark for 48 h. Fluorescent images were captured with a confocal laser-scanning microscope (LSM 880 NLO, Carl Zeiss, Germany).

To check the transcription activation activity of BnPHO2 protein, the CDS of *BnPHO2* was cloned into pGBKT7, and then transformed into the yeast strain Y2HGold according to the instructions of Matchmaker Gold Yeast Two-Hybrid system (Clontech, Japan) [24]. The protein interactions were detected on SD/-Trp, SD/-Trp/-His, SD/-Trp/-Ade, and SD/-Trp/-His/-Ade media.

### 2.6. Measurements of the Physiological Indicators

The kits related to the determination of Pi content and acid phosphatase (ACPase) activity were purchased from Komin Biotechnology (Suzhou, China). The determination of anthocyanin and chlorophyll content was based on previous reports with slight modifications [32,33]. Each experiment was performed with five biological replications.

Measurement of anthocyanin content: Add 300 μL of 1% HCl–methanol mixture to 0.01 g of material and incubate overnight at 4 °C. The next day, 200 μL ddH_2_O and 200 μL chloroform were added and centrifuged at 12,000 rpm for 5 min; then, 200 μL supernatant was aspirated, and absorbance values at 657 nm and 530 nm were measured with an enzyme-labeled instrument (Tecan INFINITE 200 PRO, Austria). Calculation: anthocyanin content (nmol/g FW) = (A_530_ − 0.33 A_657_)/plant fresh weight.

Determination of chlorophyll content: Weigh 0.04 g of leaves, add 250 μL of acetone, and leave at room temperature for 10 min. Add 8.75 mL of 80% acetone, dark-treated and placed on a shaker at 25 °C, 250 rpm overnight to extract. After the leaves turned white the next day, 1 mL of 80% acetone was added and centrifuged at 6000 g for 15 min at 4 °C. Then, 200 μL of supernatant was carefully aspirated, 80% acetone was used as blank control, and the absorbance at wavelengths 646 nm and 663 nm was measured by an enzyme-labeled instrument. Calculation: C_a_ = 12.21 A_663_ − 2.81 A_646_; C_b_ = 20.13 A_646_ − 5.03 A_663_; C_T_ = C_a_ + C_b_; Chlorophyll content (mg/g FW) = (C_T_ × V_T_)/(FW × 1000) × n. C_a_, C_b_: chlorophyll a, b concentration (mg/L); C_T_: total chlorophyll concentration (mg/L); V_T_: volume of extraction solution, 10 mL; FW: fresh weight of sample (g); n: dilution time.

### 2.7. Statistical Analyses

Statistical analyses were performed using SigmaPlot 12.5. The measurement data are expressed as mean ± SD form. ** (*p* < 0.01) and * (*p* < 0.05) represent significant differences compared to the control.

## 3. Results

### 3.1. Acquisition of Overexpressed Material

Plants overexpressing Bna-miR399c and *BnPHO2* were obtained using a *B. napus* hypocotyl genetic transformation system. The positive identification and expression assay of the target genes are shown in Figure S1. Several strains with high expression of the target gene were selected separately for self-breeding and used in subsequent experiments.

### 3.2. Overexpression of Bna-miR399c Increased the Tolerance to Low Pi Stress at Seedling Stage

To understand the biological function of miR399c in response to low Pi stress in *B. napus*, the seeds of overexpressed miR399c T_2_ plants and control J9 were selected for germination, and then the materials with consistent growth were selected for low Pi (1 μM) treatment to evaluate their resistance to low Pi at seedling stage.

**Figure 1:**
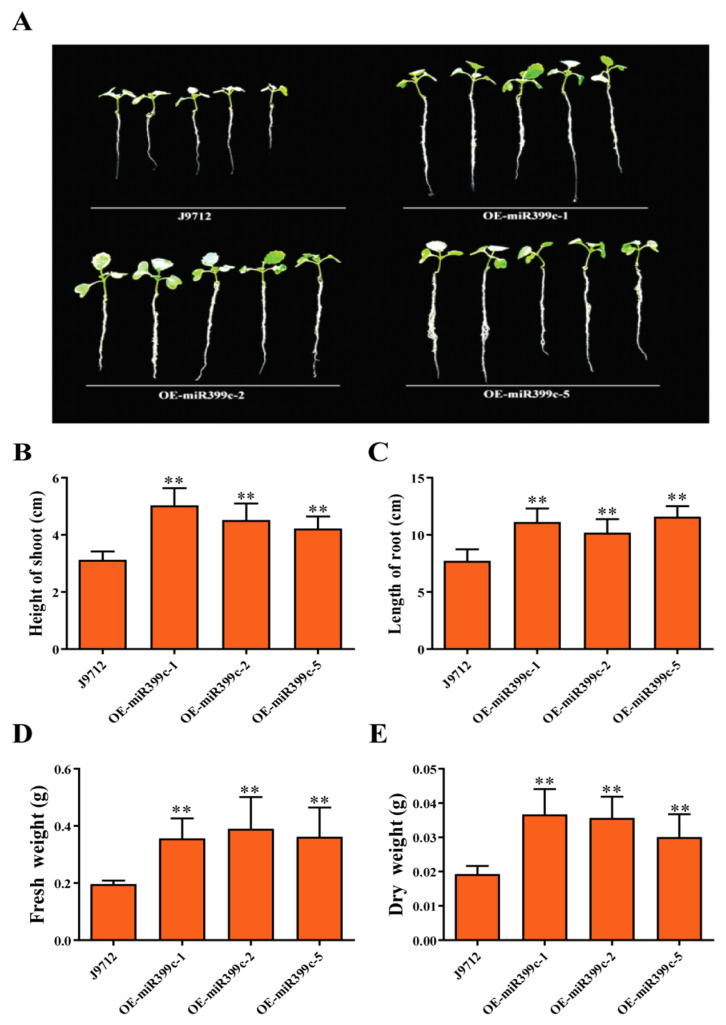
Phenotypic identification and analysis of miR399c overexpressing plants under 1 μM Pi treatment. (**A**) Growth of miR399c overexpressing plants. (**B**–**E**) Shoot height, root length, fresh weight, and dry weight of miR399c overexpressing plants. ** indicates the significant difference at *p* ≤ 0.01 level.

**Figure 2:**
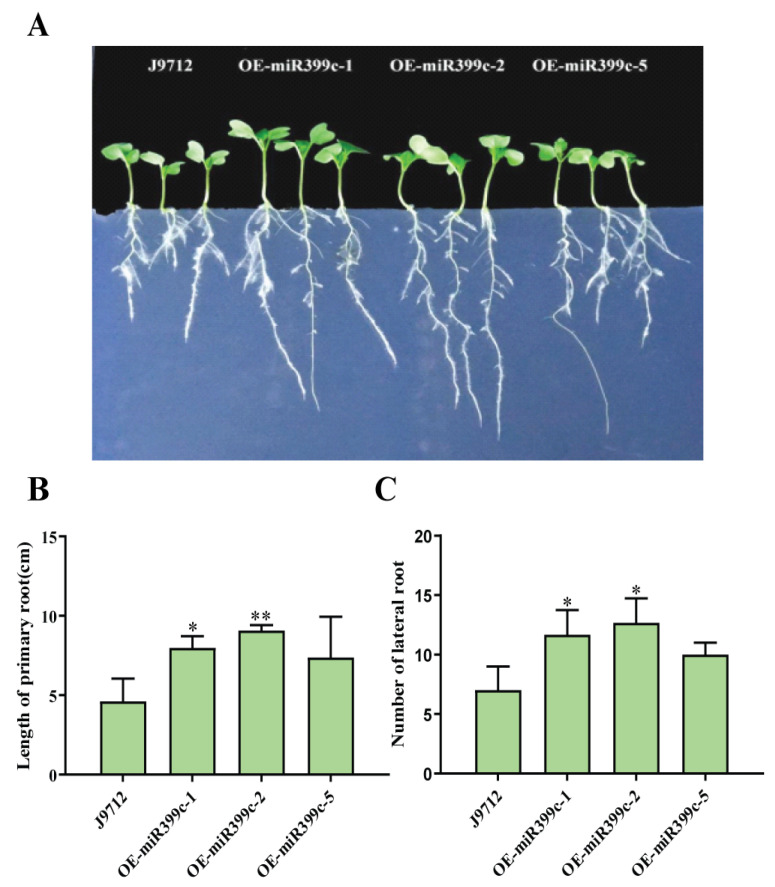
Root phenotype identification and analysis. (**A**) Root growth of miR399c overexpressing plants in 1 μM Pi solid medium. (**B**,**C**) Primary root length and number of lateral root of miR399c overexpressing plants. * indicates the significant difference at *p* ≤ 0.05 level; ** indicates the significant difference at *p* ≤ 0.01 level.

After 20 days of growth under limited Pi conditions, the growth status of Bna-miR399c overexpression material was significantly better than that of J9 (Figure 1A). The biomass, shoot height, and root length of Bna-miR399c were significantly higher than those of J9 (Figure 1B–E). In order to more intuitively observe the growth of transgenic plants and control roots under low Pi stress, we inoculated them on 1 μM Pi solid medium for treatment and observation. The results showed that Bna-miR399c overexpression material was significantly larger than J9 in both primary root length and lateral root number (Figure 2).

### 3.3. Physiological Indicators of Bna-miR399 Overexpression Plants versus Control under Low Pi Stress

**Figure 3:**
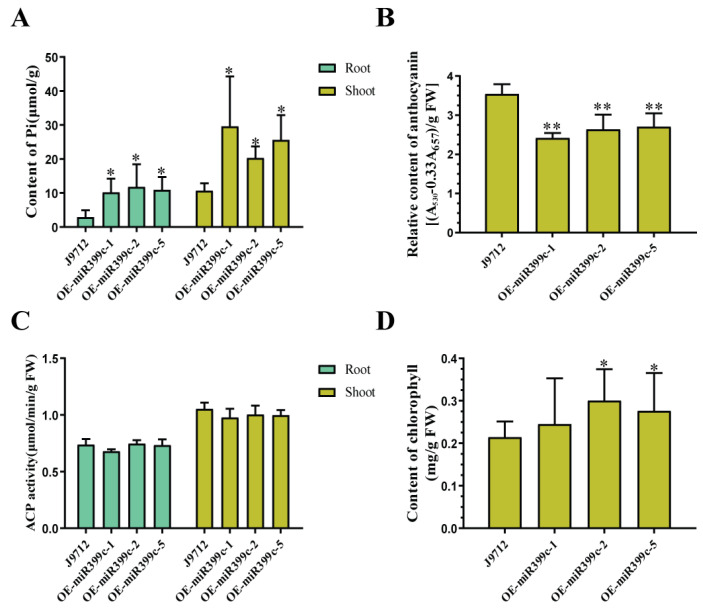
Determination of physiological indexes of miR399c overexpressing and J9 materials under low Pi stress. (**A**) Effects of low Pi treatment on Pi content in miR399c transgenic materials. (**B**) Effects of low Pi treatment on anthocyanin content in miR399c transgenic materials. (**C**) Effects of low Pi treatment on ACPase activity in miR399c transgenic materials. (**D**) Effects of low Pi treatment on chlorophyll content in miR399c transgenic materials. * indicates the significant difference at *p* ≤ 0.05 level; ** indicates the significant difference at *p* ≤ 0.01 level.

To elucidate the physiological mechanism of Bna-miR399c regulation of low Pi stress resistance in *B. napus*, we measured some physiological parameters, such as inorganic phosphorus, anthocyanin, chlorophyll content, and acid phosphatase activity, in the leaves of plants of both materials under low Pi conditions (Figure 3A–D). Under low Pi conditions, the Pi content as well as chlorophyll content of Bna-miR399c overexpressing materials in both aboveground and root tissues were significantly more than the control (Figure 3A,D). We inferred that overexpression of Bna-miR399c promoted Pi uptake, loading, and upward transport in *B. napus* compared with CK, and increased chlorophyll synthesis for normal plant life activities. Meanwhile, anthocyanin content was higher in CK plants under low Pi treatment, while anthocyanin content was significantly lower in overexpression of Bna-miR399c material (Figure 3B), indicating that overexpression of Bna-miR399c could reduce the effect of Pi environment by decreasing anthocyanin accumulation.

When plants are deficient in P, they usually hydrolyze the Pi in the soil by increasing the secretion of ACP in the roots and converting it into Pi form for plant use. We found that there was no significant difference in ACPase activity between the roots and aboveground parts of Bna-miR399c plants overexpressing Bna-miR399c under low Pi treatment and CK (Figure 3C). Combined with the difference in Pi content between the two materials, this suggests that overexpression of Bna-miR399c does not reduce sensitivity to Pi stress through the pathway of increasing ACPase activity and thus increasing the utilization of organic P in the soil, but mainly by increasing the uptake of Pi transport.

### 3.4. Upregulated Expression of Phosphorus Transport-Related Genes in Plants Overexpressing Bna-miR399c under Low Pi Stress

**Figure 4:**
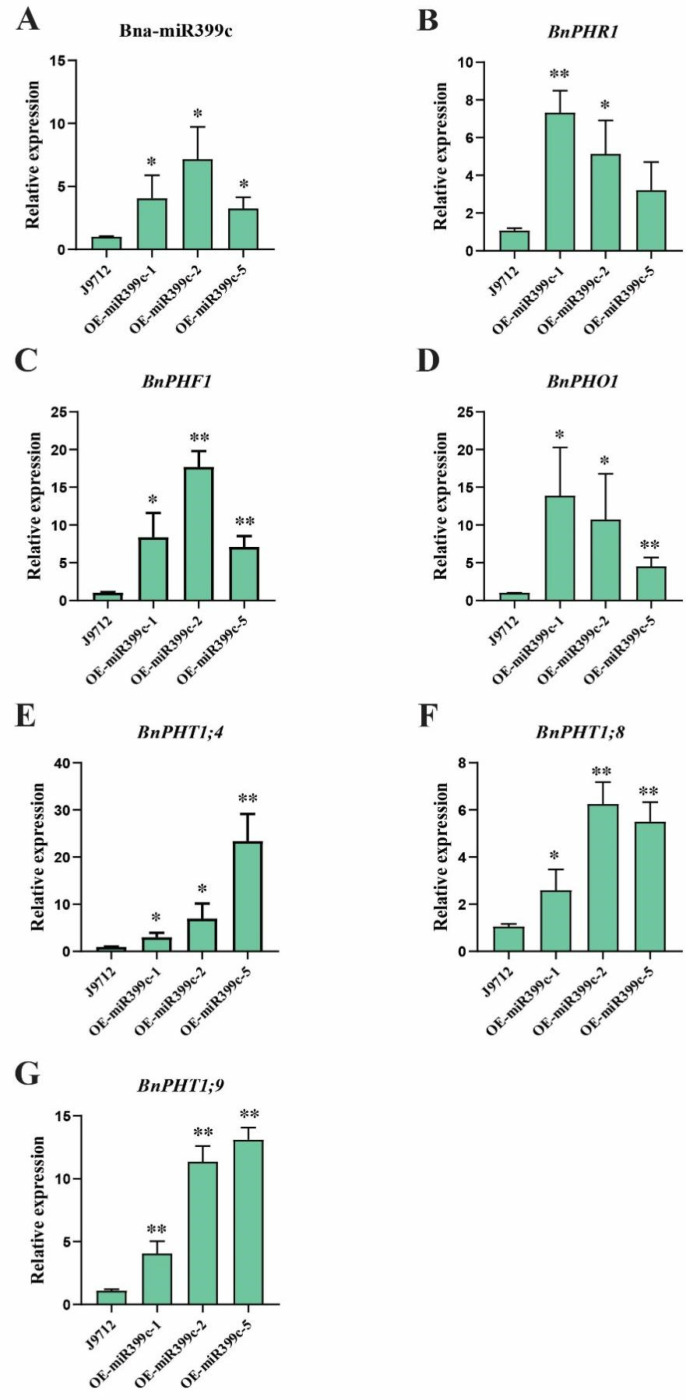
Expression analysis of P transport-related genes in miR399c overexpressing plants. (**A**) Expression of Bna-miR399c in miR399c overexpressing plants. (**B**) Expression of *BnPHR1* in miR399c overexpressing plants. (**C**) Expression of *BnPHF1* in miR399c overexpressing plants. (**D**) Expression of *BnPHO1* in miR399c overexpressing plants. (**E**) Expression of *BnPHT1;4* in miR399c overexpressing plants. (**F**) Expression of *BnPHT1;8* in miR399c overexpressing plants. (**G**) Expression of *BnPHT1;9* in miR399c overexpressing plants. * indicates the significant difference at *p* ≤ 0.05 level; ** indicates the significant difference at *p* ≤ 0.01 level.

To further explore the molecular mechanism of Bna-miR399c in response to low Pi stress, the transcript abundance of Bna-miR399c and six phosphorus transport-related genes (*BnPHR1*, *BnPHF1*, *BnPHO1*, and three members of the *BnPHT1* family) were examined in Bna-miR399c overexpression and J9 plants under low Pi (Figure 4). The results showed that the expression levels of *BnPHR1*, *BnPHF1*, *BnPHO1*, *BnPHT1;4*, *BnPHT1;8*, and *BnPHT1;9* were significantly higher than J9 due to the overexpression of Bna-miR399c (Figure 4B–G), indicating that Bna-miR399c may, by promoting the increased expression abundance of P transport-related genes and increasing the uptake and translocation of Pi nutrients, confer greater resistance to low Pi conditions in *B. napus*.

### 3.5. BnPHO2 Is the Target of Bna-miR399c

**Figure 5:**
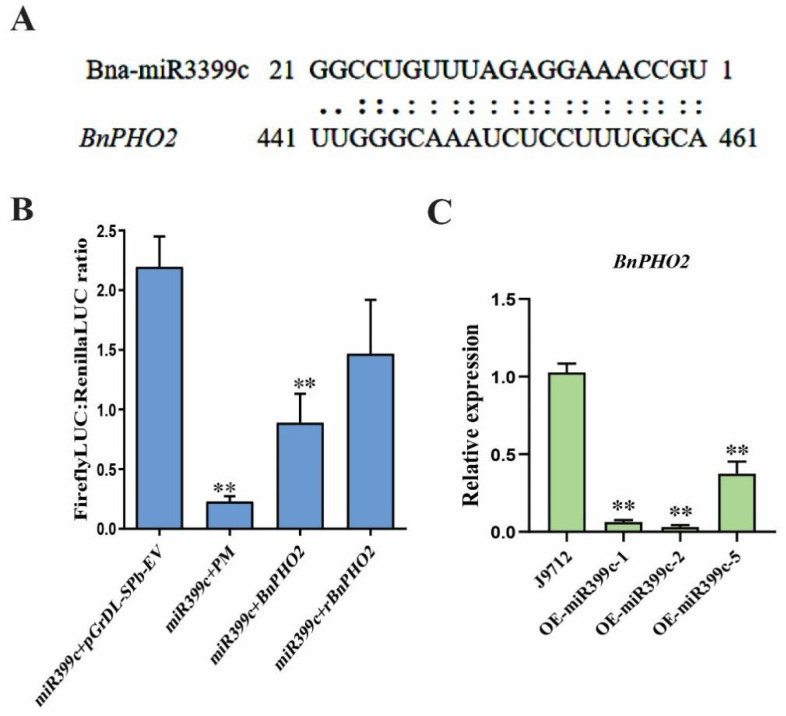
Identification of the target of miR399c. (**A**) Sequences of mature miR399c and the potential target mRNA in *B. napus*. (**B**) Validation of the regulation of miR399c and its target genes using dual-luciferase system. (**C**) Expression of *BnPHO2* in miR399c transgenic plants after low Pi treatment. ** indicates the significant difference at *p* ≤ 0.01 level.

miRNAs play a role by regulating the expression of target genes. We used the psRNATarget website (http://plantgrn.noble.org/psRNATarget/, accessed on 10 September 2022) to analyze the target genes of miR399c in *B. napus*, and the results showed that *BnPHO2* (BnaA04g19750D) is one of the possible targets. The base pairing between the mature sequence of Bna-miR399c and the target site of *BnPHO2* mRNA sequence is shown in Figure 5A. To further confirm that Bna-miR399c targets *BnPHO2*, we used a dual-luciferase reporter system to analyze the interaction between miRNA and target genes. When the *BnPHO2* potential target sequence was present in the vector, the firefly LUC reporter gene was repressed and the ratio of firefly LUC to renilla LUC was significantly lower than that of the control, which could confirm that Bna-miR399c mediated the repression of BnPHO2 expression (Figure 5B). The expression of *BnPHO2* was significantly downregulated in Bna-miR399c overexpression plants under low Pi conditions (Figure 5C). The above results indicate that Bna-miR399c in *B. napus* regulates the uptake and transport of Pi in plants under low Pi conditions by targeting the inhibition of BnPHO2 expression, and thus adapts to low Pi habitats.

### 3.6. Localization and Transcriptional Activation Ability of BnPHO2 Protein

**Figure 6:**
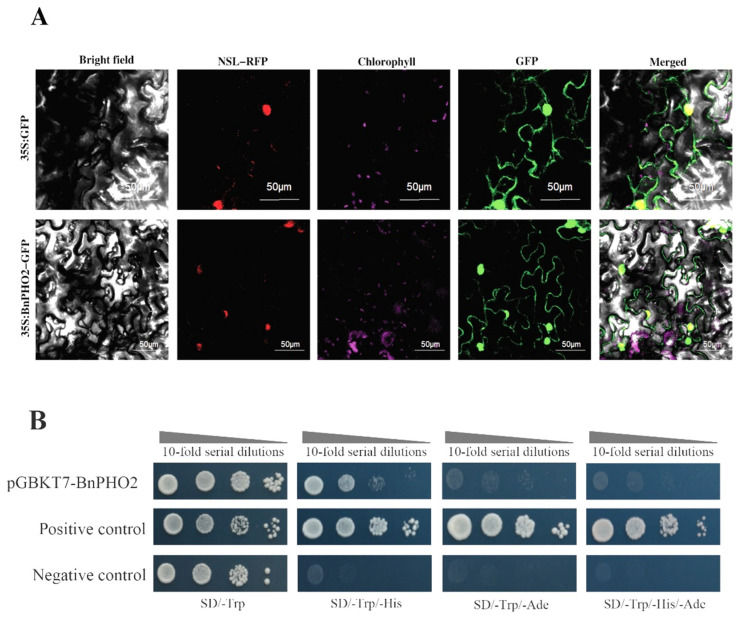
Subcellular localization and transactivation assay of BnPHO2 protein. (**A**) The constructs 35S:GFP and 35S:BnPHO2-GFP were co-expressed with the nucleus marker NLS-RFP in *N. benthamiana*. (**B**) Transactivation assay of BnPHO2 protein in yeast cells.

To clarify the site of action of the Bna-miR399c target protein BnPHO2 in plant tissue cells, we constructed the subcellular localization vector pMDC83-*BnPHO2* of the C-terminal fused GFP target protein and transformed it into tobacco, which showed that GFP signals were observed in the cytoplasm and nucleus of tobacco cells (Figure 6A). The full-length sequence of *BnPHO2* fused to the GAL4 DNA binding domain in yeast strain Y2H Gold was then used for self-activation analysis. Yeast transformants containing the pGBKT7-*BnPHO2* construct grew well on SD/-Trp medium, whereas growth was significantly inhibited in SD/-Trp/-His medium, although they were able to grow, and the growth of yeast transformants was completely inhibited on SD/-Trp/-Ade, SD/-Trp/-Ade/-His medium (Figure 6B), which showed that the full-length BnPHO2 protein had no self-activating activity.

### 3.7. BnPHO2 Is Correlated with Low Pi Tolerance in B. napus

**Figure 7:**
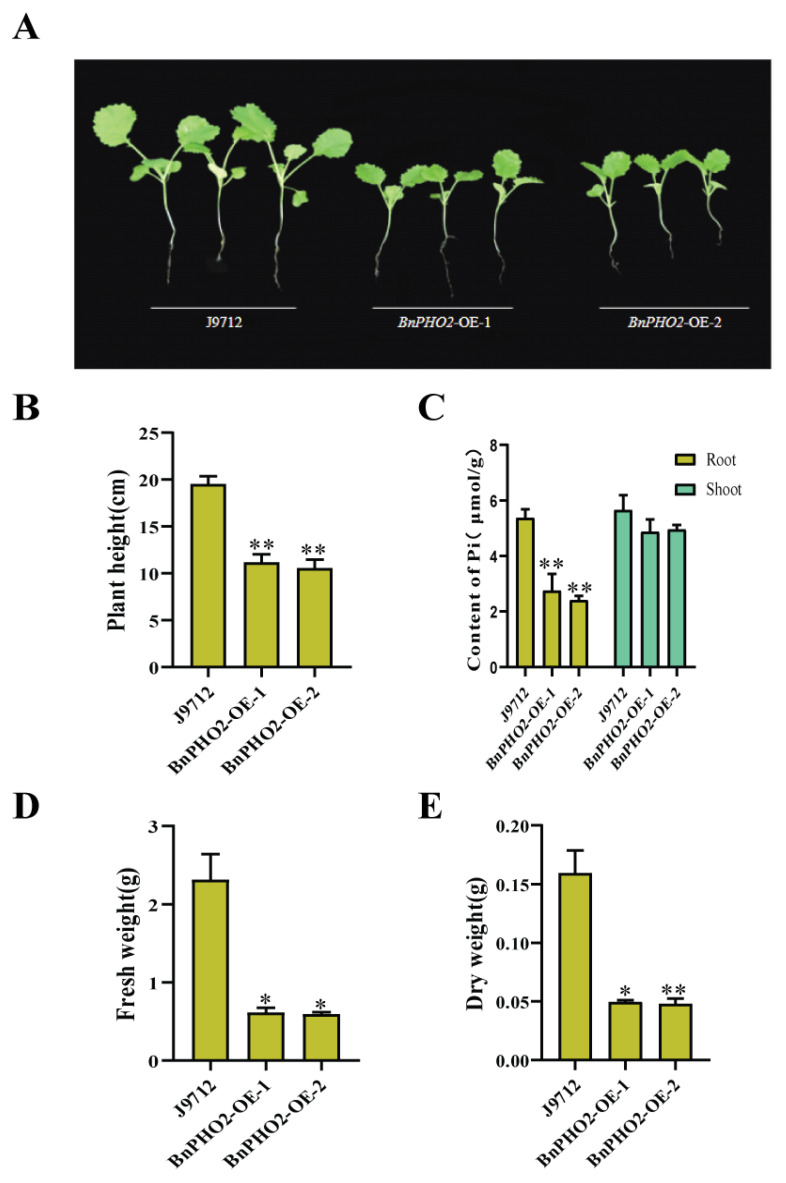
Phenotypic identification and analysis of *BnPHO2* overexpressing transgenic plants. (**A**) Growth situation of *BnPHO2* overexpressing plants. (**B**) Statistics of height of *BnPHO2* overexpressing plants. (**C**) Statistics of Pi content of *BnPHO2* overexpressing plants. (**D**) Statistics of fresh weight of *BnPHO2* overexpressing plants. (**E**) Statistics of dry weight of *BnPHO2* overexpressing plants. * indicates the significant difference at *p* ≤ 0.05 level; ** indicates the significant difference at *p* ≤ 0.01 level.

To investigate the biological function of Bna-miR399c target gene *BnPHO2*, *BnPHO2* overexpression plants were incubated with J9 plants under normal conditions for 20 days. It was found that *BnPHO2* overexpression plants were significantly lower than J9 material in terms of plant height and biomass (Figure 7A,B,D,E). Subsequently, we examined the Pi content of its above-ground and root tissues and found that the Pi content in both root and above-ground tissues of the J9 material was significantly higher than that of the overexpressing material (Figure 7C). Therefore, we concluded that overexpression of *BnPHO2* could inhibit Pi uptake and prevent its translocation from plant roots to the aboveground.

Combined with the results of Bna-miR399c, miR399c-*PHO2* module plays a key regulatory role in low Pi response in *B. napus*.

## 4. Discussion

Phosphorus plays an irreplaceable and critical role in plant growth and development. A series of complex P signaling response mechanisms have evolved in plants to counteract the damage caused by an adverse natural environment, including when plants are exposed to Pi deficiency. miR399-*PHO2* module is one of the Pi deficiency response mechanisms, which is important to ensure the normal development of plants and resist abiotic stresses, and has been reported in *Arabidopsis*, rice, maize, and other plants [2,19,34]. However, it has not been studied in *B. napus*, so this study initially explored the function of miR399c-*PHO2* in regulating Pi homeostasis in *B. napus*, which provides a necessary basis for the creation of new low-Pi-tolerant varieties of rapeseed to reduce the use of chemical fertilizers and improve agricultural economic efficiency.

The phenotypic analysis of plant biomass and other phenotypes of Bna-miR399c overexpressing plants under low Pi stress indicated that Bna-miR399c was indeed involved in the response of *B. napus* to low Pi stress and improved plant tolerance to low Pi conditions (Figure 1). Under low Pi conditions, the aboveground Pi content of the Bna-miR399c overexpression material was significantly increased compared to CK (Figure 3A), indicating that overexpression of Bna-miR399c could enhance Pi transport in *B. napus*, a result similar to related studies in *Arabidopsis* [2,35]. Interestingly, we found that the Pi content in the roots of overexpression plants was also significantly greater than that of CK under low Pi conditions, whereas overexpression of miR399c in *Arabidopsis* did not cause significant accumulation of Pi in the roots, and combined with the results on the phenotypic aspects of transgenic rapeseed roots (Figure 2), we suggest that in addition to the ability of Bna-miR399c to target *BnPHO2* to positively regulate the translocation of Pi to aboveground parts, there should be other targets in rapeseed that regulate root development, thus promoting root elongation and lateral root formation at the low Pi stage and enhancing Pi uptake to jointly cope with the low Pi environment and maintain normal life activities of the plant.

In tobacco [13], barley [17], and strawberry [36], miR399-targeted cleavage of *PHO2* gene expression was demonstrated with the help of the 5′RLM-RACE technique. In the present study, we also found that Bna-miR399c targeted to cut *BnPHO2* using a dual-luciferase reporter system (Figure 5B). The results of qRT-PCR showed downregulated expression of *BnPHO2* in Bna-miR399c overexpression plants (Figure 5C), and these results demonstrated that *BnPHO2* is one of the target genes of Bna-miR399c in *B. napus*. It has been shown that *PHO2* encodes a ubiquitin-binding enzyme E2, which regulates P uptake, transport, and utilization to balance Pi homeostasis in plants by mediating the degradation of P transport proteins such as PHO1 and PHT1 through ubiquitination [37,38]. PHT1 is present in the epidermis and cortex of the plant root system and its main function is to absorb Pi from the soil into the root, while PHO1 is present in the vascular tissue of the root and is responsible for loading Pi into the xylem vascular bundle and transferring it to the plant above ground for plant use [39,40]. miR399 expression abundance increases in the presence of Pi restriction, and at the 5′-UTR region of the site, cleaves it, which in turn inhibits *PHO2* expression, thereby unlocking the ubiquitinated degradation of *PHO1* and *PHT1* by *PHO2* and increasing the uptake and translocation of Pi. The results of this study show that under low Pi conditions, overexpression of Bna-miR399c rapeseed plants showed downregulated expression of the target gene *BnPHO2* and upregulated expression of P transport-related genes such as *PHO1* and *PHT1*, which improved the loading and upward transport of Pi in the plants, while overexpression of *BnPHO2* plants showed the opposite phenotype (Figure 7A).

Combining the experimental results of this study with previous reports in other crops, we propose a hypothetical pathway for the involvement of miR399 in low Pi stress in *B. napus*. Upon low Pi stress, *BnPHR1* was activated and responded rapidly with enhanced transcriptional activity to stimulate the Pi response pathway. Then, *BnPHR1* was regulating the expression of Bna-miR399c. The expression of the target gene *BnPHO2* was suppressed after the enhancement of miR399c activity, and the ubiquitination degradation was inhibited, which in turn promoted the expression of *PHO1* and *PHF1*. On the one hand, the enhanced transcriptional activity of *PHF1* activated the PHT1 family of P transport proteins, thus promoting the uptake of external Pi; on the other hand, the increased expression of *PHO1* abundance also promoted the transport of Pi between plant tissues. Under the joint regulation of Pi uptake and transport response mechanisms, the tolerance of rapeseed to a low Pi environment is enhanced to maintain its normal growth process. In addition, in the process of plant involvement in Pi stress, phosphorus signaling does not act alone, and its relationship with other signaling pathways still needs to be investigated in subsequent experiments.

## 5. Conclusions

P is one of the essential mineral elements for plants. We investigated the homeostatic regulatory mechanism of the miR399-*PHO2* module involved in Pi stress in *B. napus*. The results showed that miR399c-*PHO2* was involved in the response of rapeseed to low Pi stress, and overexpression of Bna-miR399c could suppress the expression of *BnPHO2* and inhibit the ubiquitination of P transport proteins, which in turn promoted Pi uptake and transport and showed better adaptation to the low Pi environment.

## Data Availability

Not applicable.

## Supplementary Materials

The following supporting information can be downloaded at: https://www.mdpi.com/article/10.3390/life13020310/s1, Table S1: Primers used for qRT-PCR; Figure S1: PCR identification of T0 generation of transgenic plants.
