# Supplementary material for: Functional Analysis of Bna-miR399c-PHO2 Regulatory Module Involved in Phosphorus Stress in Brassica napus
Source: Life (Basel). 2023 Jan 22;13(2):310. doi: 10.3390/life13020310 (PMC9965056; doi:10.3390/life13020310)
Supplement: Supplementary file 1 [file life-13-00310-s001.zip › Figures S1.pdf]

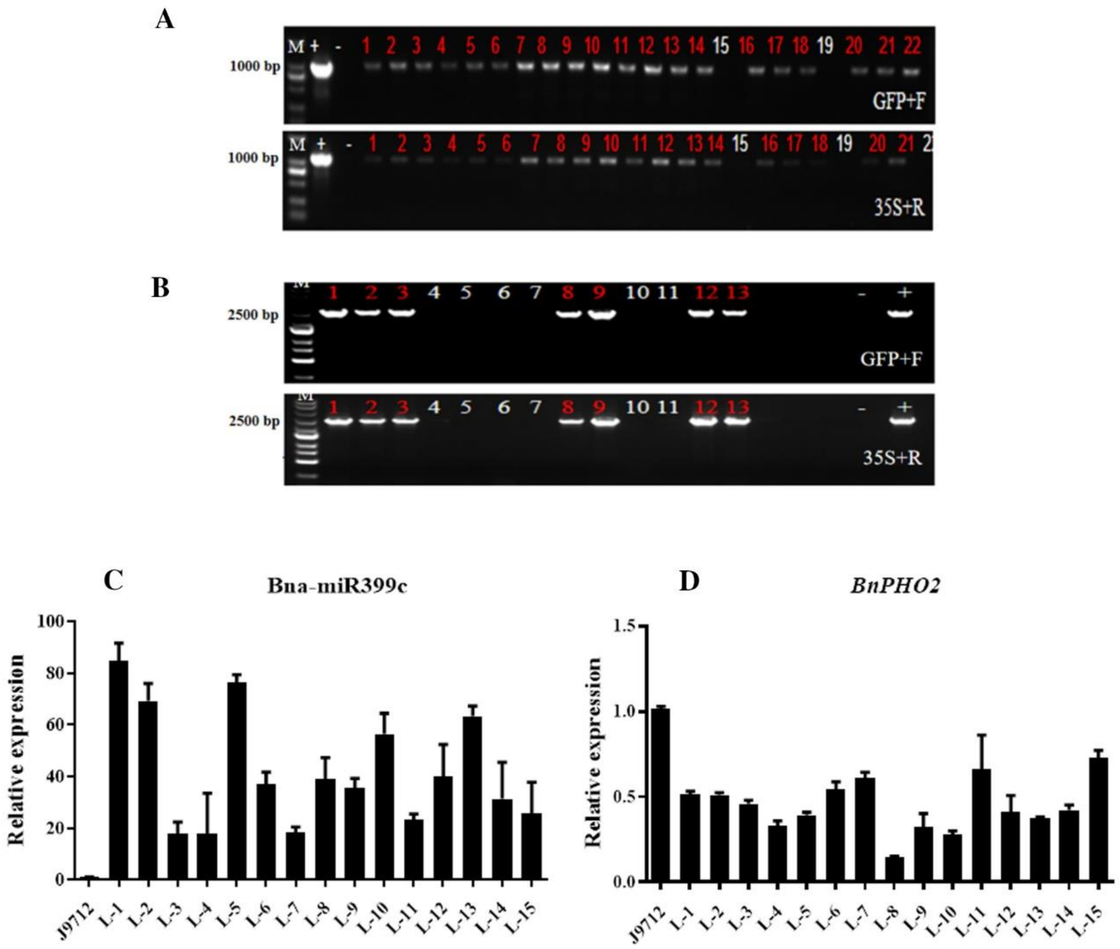

Figure S1 **PCR identification of T_0 generation of transgenic plants**

A: PCR identification of OE-Bna-miR399c transgenic plants, M: DL2501 DNA marker, +: Positive control, -: Negative control, 1-22: Transgenic plants (red: positive, white: negative); B: PCR identification of OE-*BnaPHO2* transgenic plants, M: DL2504 DNA marker, +: Positive control, -: Negative control, 1-13: Transgenic plants (red: positive, white: negative).
